# Comparative Analysis of Surgical Outcomes Between Open Herniotomy and Laparoscopic Hernia Repair Among Pediatric Patients with Inguinal Hernias: A Five-Year Retrospective Review at Tanzania's National Hospital

**DOI:** 10.24248/eahrj.v9i1.832

**Published:** 2025-09-30

**Authors:** Satrumin Shirima, Daniel Kitua, Ally Mwanga, Nashivai Kivuyo, Anab Issa, Innocent Kileo, Jeanine Justiniano, Meshack Brighton, Mohammed Salim

**Affiliations:** a Department of Surgery, Muhimbili University of Health and Allied Sciences, Dar es Salaam, Tanzania; b Muhimbili University of Health and Allied Sciences, Dar es Salaam, Tanzania; c Mwanza Intervention Trials Unit, National Institute of Medical Research, Mwanza, Tanzania; d Department of Surgery, University of California Medical Center, Sacramento, CA, United States; e Pediatric Surgery Unit, Muhimbili National Hospital, Dar es Salaam, Tanzania

## Abstract

**Background:**

Inguinal hernias (IH) are common congenital anomalies in children, requiring surgical repair to prevent complications. The primary methods for repairing pediatric IH are laparoscopic hernia repair (LHR) and open herniotomy (OH). However, there is limited evidence comparing postoperative outcomes between these approaches in Low- and Middle-Income Countries (LMICs).

**Objective:**

To compare postoperative outcomes between OH and LHR among pediatric patients treated at Muhimbili National Hospital (MNH) in Tanzania.

**Methodology:**

This 5-year retrospective comparative cohort study included 156 pediatric patients under 14 years who underwent OH or LHR at MNH from January 2019 to December 2023. Participants were selected using a hybrid random-consecutive sampling technique. LHR and OH patients were recruited at approximately a 1-to-4 ratio. Data was collected from patient records and via phone interviews. Inferential statistical tests were used to compare the outcomes of the two surgical methods.

**Results:**

Participants had a median age of 19.5 months (IQR 10–36) at presentation, with the majority being male (142, 91%). The median age was slightly higher in the LHR group [21.5 (IQR 12–46.5)] compared to 19 months (IQR 9–36) in the OH group. In the subgroup analysis of bilateral hernias (n=22), measures such as the duration of surgery, postoperative length of stay, total hospitalization time, parents` satisfaction, and cosmetic rating favored LHR (*p*<.05). Satisfaction and cosmesis were also better with LHR for unilateral hernias (n=134) (*p*<.05). However, the overall recurrence rate was higher with unilateral LHR at 22.7% (5/17) compared to 4.5% (5/107) with OH, *p*<.05.

**Conclusion:**

While LHR demonstrated shorter operative time, faster recovery, and favourable cosmetic outcomes, the potential for hernia recurrence warrants careful consideration when selecting the surgical approach. Given the inherent methodological limitations, these findings should be interpreted with caution. Nevertheless, the findings provide valuable preliminary evidence that can inform future larger-scale, well-powered studies aimed at evaluating the long-term outcomes of LHR in LMIC settings.

## BACKGROUND

Inguinal hernias are one of the most common congenital anomalies in pediatric patients, necessitating surgical repair. Pediatric inguinal hernias are typically indirect, involving the protrusion of intra-abdominal contents through a patent processus vaginalis (PPV). The incidence of primary inguinal hernia varies, with approximately 8 to 50 cases per 1000 live births in term babies and higher incidences (>20%) in extremely low birth weight (<1000 g) and premature infants.^1–3^ However, the incidence of pediatric inguinal hernias in low-middle-income countries (LMICs) is underexplored.

Inguinal hernias can lead to several complications, including bowel incarceration, with an estimated incidence of 31% in the pediatric population.^4^ The relatively high incidence of complications prompts the need for surgical repair, which utilises two main approaches: open herniotomy and laparoscopic hernia repair.^5^ Open high ligation of the hernia sac is the traditional gold standard. However, laparoscopic repair emerged as an alternative since it was first described by Montupet in 1993, and its use has increased fivefold in the past decade.^6^ The most common technique used for laparoscopic repair is the Needle-Assisted Repair of Inguinal Hernia (LNAR).^7^ The LNAR is a minimally invasive procedure performed through a single umbilical incision, where a needle is used to place a purse-string suture around the internal inguinal ring. While LNAR offers the benefits of a minimally invasive approach, its direct costs may be higher than open repair due to the need for disposable instruments and operating room resources.^8^

In experienced centres, both approaches yield comparable outcomes.^5,9–13^ However, despite the increasing evidence, several settings still have controversy regarding the best approach for pediatric hernia management.^14^ The steep learning curve of minimally invasive surgery (MIS) in LMICs, where the availability of such services is limited, promotes further exploration of these approaches. Moreover, most studies in LMICs comparing hernia repair techniques have focused on adult patients, underexploring pediatric cases. Therefore, this study aimed to compare the outcomes of laparoscopic and open hernia repair in pediatric patients at a quaternary hospital in Tanzania, evaluating their impact on the overall treatment outcomes.

## METHODS

### Study Design, Setting, and Population.

We conducted a 5-year retrospective comparative cohort study in March 2024 among pediatric patients aged less than 14 years who underwent inguinal hernia repair surgery at the Pediatric Surgery Unit of Muhimbili National Hospital, Tanzania, between 2019 and 2023. The Unit is equipped to provide quaternary surgical services and serves as the national referral centre, with a catchment area encompassing the entire country under the referral-based healthcare system. The centre has five years of experience performing pediatric laparoscopic procedures, including hernia repairs. The commonly used technique for laparoscopic hernia repair is LNAR, while the technique utilised for open hernia repair is high ligation and excision of the hernia sac. Most of the procedures are performed by pediatric surgeons. Before performing this study, ethical approval was obtained from the Muhimbili University of Health and Allied Sciences Ethical Review Board in September 2023 (Ref. DA.282/298/01L/620).

### Sample Size Estimation and Sampling Technique

Due to the retrospective nature of the study and the limited number of eligible cases, the sample size was pragmatically determined by including all available patients who underwent LHR during the study period. To enhance statistical power, all eligible laparoscopic cases were included consecutively. For the open repair group, where the patient pool was larger, a simple random sampling method was used to select participants at a ratio of 1:4 relative to the laparoscopic group. This approach aimed to maximise the study's ability to detect a statistically significant difference in outcomes between the two surgical techniques.

### Data Collection Procedures

Data was collected using a structured checklist that included the socio-demographic characteristics of the patients, such as the child's age and sex at presentation.

Clinical characteristics recorded included gestational age at birth, localisation and type of the hernia, surgical indication (elective or emergent), the status of associated medical or surgical conditions, the timing of the surgery, length of surgery, and length of hospital stay. Additional information gathered included complications such as wound hematoma, surgical site infection, reactive hydrocele, scrotal edema, scrotal hematoma, and overall recurrence between 3 months and 5 years. These details were extracted from the medical record. The satisfaction, cosmetic rating, and presence of recurrence were obtained from follow-up clinic notes or, when these were unavailable, through structured telephone interviews with the parent or guardian. Follow-up duration ranged from 3 months to 5 years, with telephone interviews conducted in April 2024. This combined data collection strategy enhanced the completeness of data and ensured the capture of outcome measures that were not consistently documented in the medical records.

### Data Management and Statistical Analysis

The collected data were analysed using the Statistical Package for Social Sciences version 22. Descriptive statistics summarised the characteristics of the study population: categorical variables were summarised using frequencies and proportions. In contrast, continuous variables were summarised using means and standard deviations or medians and interquartile ranges, depending on the distribution. The groups were compared using the chi-square test, Fisher's exact test, or an independent t-test, depending on the specific data requirements. A *P* value of less than .05 was considered statistically significant for interpreting the results. Given the retrospective, exploratory design and pragmatic sample size, no formal adjustment for multiple comparisons was performed.

### Ethics Approval and Consent to Participate.

Ethical clearance was obtained from the Muhimbili University of Health Allied Sciences (MUHAS) ethical committee with reference number (Ref. DA.282/298/01L/620). We requested a waiver of consent to collect data from the patient's files; however, consent was obtained from all patients who were contacted over the phone for data collection.

## RESULTS

### Sociodemographic and clinical characteristics of children who underwent inguinal hernia repair

**FIGURE 1:**
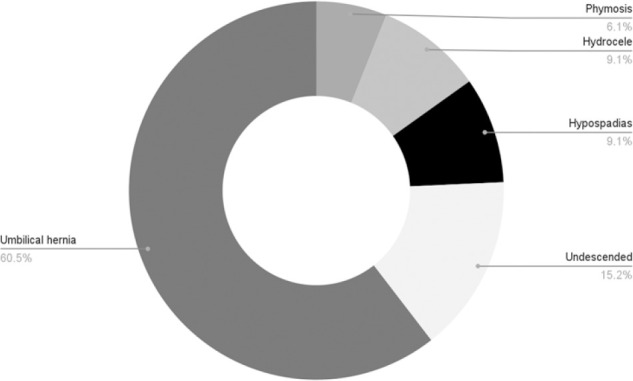
Surgical Co-Morbidities Among Children Who Underwent Inguinal Hernia Repair

**FIGURE 2:**
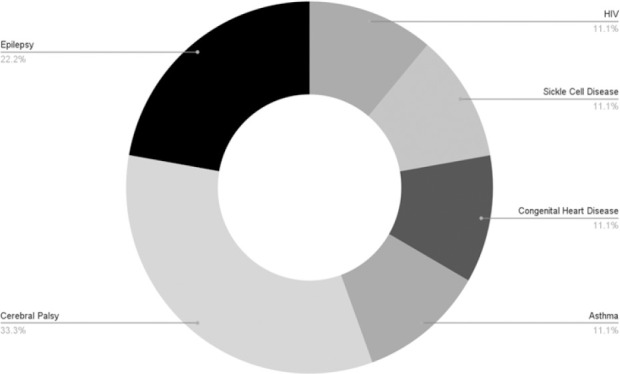
Medical Co-Morbidities Among Children Who Underwent Inguinal Hernia Repair

Table 1 illustrates that out of 156 children who underwent inguinal hernia repair, the majority were males, accounting for 91%, with most aged between 1 and 5 years. The median age at presentation was 19.5 months (IQR 10–36). Only 30 children (19.2%) underwent laparoscopic hernia repair, with approximately four open herniotomy cases included per LHR case for the comparative analysis. Out of all cases, 95.5% were for first-time hernia repairs. Hernias were predominantly right-sided at presentation (61.8%), and bilateral disease was observed in 18.9%. Most children presented with a reducible hernia (87.5%). Additionally, 33 children (21.2%) had other associated surgical conditions, umbilical hernia being the most common, occurring in 20 (60.5%) of these cases, as depicted in Figure 1. A small number of children, 10 (6.4%), had chronic medical conditions, cerebral palsy being the most prevalent (33.3%), as summarized in Figure 2.

**TABLE 1:** Social Demographics and Clinical Characteristics of Children Who Underwent Inguinal Hernia Repair Variable LHR (N=30) OH (N=126)

Variable	LHR (N=30)	OH (N=126)	*P* Value
Sex			
Male	29 (96.7%)	113 (89.7%)	.308
Female	1 (3.3%)	13 (10.3)	
Age			
<6 months	1 (3.3%)	15 (11.9%)	.269
6 months-1 year	9 (30.0%)	33 (26.2%)	
1 year-5 years	16 (53.3%)	71 (56.3%)	
>5 years	4 (13.3%)	7 (5.6%)	
Gestational age at birth (weeks)			
<37 weeks	5 (16.7%)	22 (17.5%)	.918
≥37 weeks	25 (83.3)	104 (82.5%)	
Median age at presentation (months)	21.5 (IQR 12–46.5)	19 (IQR 9–36)	.632
Side of the hernia			
Right	19 (63.3%)	76 (60.3%)	.023
Left	3 (10.0%)	36 (28.6%)	
Bilateral	8 (26.7%)	14 (11.1%)	
Type of hernia			
Reducible	27 (90.0%)	107 (84.9%)	.715
Incarcerated	3 (10%)	10 (7.9%)	
Strangulated	0 (0%)	9 (7.1%)	
Surgical indication			
Elective	29 (96.7%)	112 (88.9%)	.306
Emergency	1 (3.3%)	14 (11.1%)	
Hernia surgery history			
First hernia	28 (93.3%)	121 (96.0%)	.620
Recurrent hernia	2 (6.7%)	5 (4.0%)	
Presence of surgical co-morbidities			
Yes^a^	8 (26.7%)	25 (19.8%)	.411
No	22 (73.3%)	101 (80.2%)	
Presence of medical co-morbidities			
Yes^b^	4 (13.3%)	6 (4.8%)	.101
No	26 (86.7%)	120 (95.2%)	

a Refer to Figure 1

b Refer to Figure 2

Key:
LHR-Laparoscopic Hernia Repair; OH-Open Herniotomy.

### Comparison of Laparoscopic Hernia Repair and Open Herniotomy Repair Among Children Who Underwent Inguinal Hernia Repair

Table 2 compares the outcomes between open and laparoscopic approaches for inguinal hernia repair among 156 children, categorised into bilateral and unilateral repair groups. The study found no significant difference in the duration of surgery, presence of wound complications, scrotal complications, or other examined complications between the two approaches in unilateral hernia repairs (*P*>.05). Additionally, parents expressed greater satisfaction with the laparoscopic hernia repair (*P*=.002), and the cosmetic appearance of the surgical scar was rated more favourable in the laparoscopic group (*P*=.001). However, the recurrence rate was notably higher in the laparoscopic group (22.7%) compared to the open herniotomy group (4.5%) (*P*=.011).

**TABLE 2:** Comparison Between Laparoscopic Inguinal Hernia Repair and Open Inguinal Herniotomy

Variable	Unilateral Hernia			Bilateral Hernia		
	Repair			Repair		
	LHR (N=22)	OH (N=l 12)	*P* Value	LHR (N=8)	OH (N= 14)	*P* Value
Duration of surgery in minutes (mean ± standard deviation)	63.86 ± 22.25	63.71 ± 15.63	.968	62.50 ± 7.81	111.07 ± 30.64	<.001
Wound complications						
Hematoma	0 (0.0%)	1 (0.9%)	1	0 (0.0%)	0 (0.00%)	.254
Surgical Site Infection	0 (0.0%)	4 (3.6%)		0 (0.0%)	4 (28.6%)	
None	22 (100%)	107 (95.5%)		8 (100%)	10 (71.4%)	
Scrotal complications						
Reactive hydrocele	1 (4.5%)	2 (1.8%)	.452	0 (0.0%)	3 (21.4%)	.393
Scrotal edema/orchiditis	0 (0.0%)	2 (1.8%)		0 (0.0%)	0 (0.0%)	
Scrotal hematoma	0 (0.0%)	1 (0.9%)		0 (0.0%)	1 (7.1%)	
None	21 (95.5%)	96 (85.7%)		7 (87.5%)	10 (71.4%)	
Not applicable (Female)	0 (0.0%)	11 (9.8%)		1 (12.5%)	0 (0.0%)	
Other complications						
Bowel obstruction	0 (0.0%)	1 (0.9%)	1.000	0 (0.0%)	0 (0.0%)	1.000
Nausea and Vomiting	0 (0.0%)	2 (1.8%)		0 (0.0%)	1 (7.1%)	
None	22 (100%)	109 (97.3%)		8 (100%)	13 (92.9%)	
Length of postoperative hospitalization in days (mean ± Standard Deviation)	1.68 ± 1.17	2.50 ± 2.48	.132	1.13 ± 0.35	3.43 ± 1.34	<.001
Total duration of hospitalization in days (mean ± Standard Deviation)	3.68 ± 1.67	4.32 ± 3.72	.431	2.88 ± 0.35	4.93 ± 1.98	.009
Recurrence within 3 months to 1 year (overall recurrence)						
Yes	5 (22.7%)	5 (4.5%)	.011	0 (0.0%)	0 (0.0%)	–
No	17 (77.3%)	107 (95.5%)		8 (100%)	14 (100%)	
Parent's satisfaction						
Dissatisfied	0 (0.00%)	4 (3.6%)	.002	0 (0.00%)	2 (14.3%)	.036
Neutral	2 (9.1%)	13 (11.6%)		0 (0.00%)	2 (14.3%)	
Satisfied	7 (31.8%)	77 (68.8%)		3 (37.5%)	9 (64.3%)	
Very satisfied	13 (59.1%)	18 (16.1%)		5 (62.5%)	1 (7.1%)	
Surgical scar cosmetic rating						
Bad	0 (0.00%)	3 (2.8%)	.001	0 (0.00%)	8 (57.1%)	<.001
Good	5 (22.7%)	104 (95.4%)		1 (12.5%)	6 (42.9%)	
Excellent	17 (77.3%)	2 (1.8%)		7 (87.5%)	0 (0.00%)	

Key:
LHR-laparoscopic hernia repair OH-open herniotomy.

In cases of bilateral hernia repair, the laparoscopic approach resulted in a significantly shorter operation time of 62.50±7.81 minutes than 111.07±30.64 minutes (*P*=.001), reduced length of stay postoperatively of 1.13±0.35 days compared to 3.43±1.34 days (*P*<.001), and total hospitalization duration of 2.88±0.35 days as opposed to 4.93±1.98 days (*P*=.009) for open herniotomy. However, there were no significant differences between the two groups regarding wound, scrotal, recurrence, or other complications assessed (*P*>.05). Parents also showed a preference for the laparoscopic hernia repair (*P*=.036), with better cosmetic outcomes reported for the surgical scar (*P*=.001).

## DISCUSSION

This study aimed to compare the outcomes of laparoscopic versus open hernia repair at Tanzania's National Hospital. Our results revealed that most children who underwent inguinal hernia repair were males over the age of one year, contrary to previous studies that indicated a similarly high prevalence in the male pediatric population, but with more frequent presentations occurring within the first year of life.^15–17^ Poor health-seeking behaviours and disparities in access to healthcare facilities in LMICs could explain this. Like other studies, approximately two-thirds of the hernias were right-sided, attributed to a developmental delay in the closure of the processus vaginalis during fetal development ^17,18^. The right testicle descends more slowly than the left, potentially leading to a slower closure of the processus vaginalis on the right side. Additionally, most hernias were reducible, with an incarceration and strangulation rate of 14.1%, which is within the previously reported estimates that range between 2% and 31% in the pediatric population.^4^

A substantial proportion of children underwent open herniotomy, likely due to the surgeons’ preference and its affordability when compared with laparoscopic repair, as LNAR is four times more costly than open herniotomy in our settings. This trend partly mirrors findings in other settings where open repair is more common, as many patients cannot afford the higher costs of laparoscopic surgery.^19,20^. Nonetheless, LHR may offer favorable outcomes, potentially offsetting the higher upfront expenses.^21^ The mean operative duration was comparable between the laparoscopic and open surgery groups, averaging approximately 64 minutes. However, in cases of bilateral hernia, the laparoscopic approach demonstrated significantly shorter operative time when compared to the open approach. These findings align with other studies that report no significant difference in operative time between laparoscopic and open hernia repair for unilateral cases. In contrast, the laparoscopic approach notably reduces operative time for bilateral repairs.^5,14,22^ The technical advantage of the laparoscopic approach in bilateral hernia may be attributed to the ability to utilize the same access ports to efficiently address both defects, as opposed to the open approach, which requires separate incisions.

Most parents rated the surgical scars from laparoscopic repair as excellent when compared with open repair for both unilateral and bilateral cases, consistent with findings from previous studies.^23^ However, a meta-analysis found no difference between laparoscopic and open repair in terms of wound complications such as hypertrophic scars, unsightly scars, or stitch granulomas.^14^ Similarly, parents expressed greater satisfaction with laparoscopic repairs over open repairs for both unilateral and bilateral cases, at a statistically significant level of difference. ] This increased satisfaction may be attributed to the shorter perioperative hospital stay in the laparoscopic hernia group, particularly in cases of bilateral hernia. Contrary to these findings, a systematic meta-analysis comparing laparoscopic and open hernia repair in children found no statistically significant difference in postoperative hospital stay between the two methods.^14^

Recurrence rates were higher in the laparoscopic group for unilateral hernias; however, firm conclusions are limited, as the small sample size amplifies the impact of a few cases and may overstate the actual recurrence rate. Moreover, the steep learning curve in the initial years likely contributed to the higher recurrence rates, a concern expected to resolve over time as surgeons reach the plateau phase of mastering laparoscopy. Furthermore, some recurrences were self-reported via telephone interviews rather than confirmed clinically, introducing the possibility of reporting bias. Nevertheless, reviews show minimal non-significant differences in recurrence rates between the two approaches.^24,25^ Both laparoscopic and open inguinal hernia repairs demonstrated low rates of postoperative wound, scrotal, and gastrointestinal complications in both unilateral and bilateral cases. Although open repair had slightly higher complication rates compared with laparoscopic repair, this difference was not statistically significant, consistent with findings from other studies and systematic reviews.^5,23,26^ However, some studies suggest that laparoscopic hernia repair, particularly in infants, is associated with fewer complications, a finding that merits further investigation.^24,25,27^

Policy considerations for the adoption of LHR/MIS in LMICs should focus on enhancing surgical capacity through structured training programs and establishing robust mentorship frameworks. Given the steep learning curve associated with LHR/MIS, implementing simulation-based training and incorporating laparoscopic modules into surgical curricula can facilitate skill acquisition and proficiency.^28^ Additionally, fostering collaboration between academic institutions and healthcare facilities can promote knowledge exchange and support the development of standardised surgical protocols.^29^ Investing in infrastructure to support laparoscopic surgery, such as procuring essential equipment and ensuring maintenance, is crucial for the sustainability of MIS programs. Furthermore, integrating MIS into national surgical plans and aligning with global health initiatives can bolster advocacy efforts and secure necessary resources.^29^ An additional consideration includes the need for cost-effectiveness analyses tailored to local contexts. By addressing these policy dimensions, LMICs can enhance the feasibility and sustainability of LHR/MIS, ultimately improving patient outcomes and advancing surgical care.

### Study Limitations

Despite these findings, the study's limitations include its pragmatic power based on available cases and single-center design, which may limit generalizability and the ability to detect subtle differences in outcomes. The retrospective design meant the surgical approach was chosen by surgeons, introducing potential selection bias that could influence the comparison between groups. Combining medical records with phone interviews for follow-up may have caused data heterogeneity and recall bias. Variations in follow-up duration could also impact outcome consistency. Additionally, multiple statistical comparisons increase the risk of Type I error. Nevertheless, the findings provide important preliminary evidence that can inform the design of future, larger, well-powered studies aimed at comprehensively evaluating the long-term outcomes and clinical effectiveness of LHR within LMIC settings.

## CONCLUSION

This study highlights that while laparoscopic hernia repair offers significant advantages, including shorter surgery duration, quicker recovery, and superior cosmetic outcomes, particularly in cases of bilateral hernia, it also presents an increased risk of hernia recurrence compared to open herniotomy. These findings suggest that the choice of surgical approach should be tailored to each patient, considering the immediate benefits of laparoscopic surgery and the potential for long-term complications such as recurrence.

Given the increased risk of recurrence observed with laparoscopic hernia repair, especially in unilateral hernias, it is recommended that surgeons carefully weigh the benefits of this approach against the potential for recurrence. Further large-scale, multicenter studies are necessary to identify the factors contributing to recurrence in laparoscopic hernia repair. Such research should aim to refine surgical techniques and protocols to minimize recurrence rates and improve long-term outcomes.
